# Targeting post-stroke neuroinflammation with Salvianolic acid A: molecular mechanisms and preclinical evidence

**DOI:** 10.3389/fimmu.2024.1433590

**Published:** 2024-07-30

**Authors:** Hongchun Yang, Muhammad Mustapha Ibrahim, Siyu Zhang, Yao Sun, Junlei Chang, Hui Qi, Shilun Yang

**Affiliations:** ^1^ Department of Neurosurgery, Peking University Shenzhen Hospital, Shenzhen, Guangdong, China; ^2^ Institute of Biomedicine and Biotechnology, Shenzhen Institute of Advanced Technology, Chinese Academy of Sciences, Shenzhen, Guangdong, China; ^3^ University of Chinese Academy of Sciences, Beijing, China; ^4^ Nanfang Hospital, Southern Medical University, Guangzhou, Guangdong, China; ^5^ Department of Anesthesiology, Zhujiang Hospital, Southern Medical University, Guangzhou, Guangdong, China

**Keywords:** Salvianolic acid A, ischemic stroke, anti-inflammation, neuroprotection, natural medicine

## Abstract

Salvianolic acid A (SalA), a bioactive compound extracted from Salvia miltiorrhiza, has garnered considerable interest for its potential in ameliorating the post-stroke neuroinflammation. This review delineates the possible molecular underpinnings of anti-inflammatory and neuroprotective roles of SalA, offering a comprehensive analysis of its therapeutic efficacy in preclinical studies of ischemic stroke. We explore the intricate interplay between post-stroke neuroinflammation and the modulatory effects of SalA on pro-inflammatory cytokines, inflammatory signaling pathways, the peripheral immune cell infiltration through blood-brain barrier disruption, and endothelial cell function. The pharmacokinetic profiles of SalA in the context of stroke, characterized by enhanced cerebral penetration post-ischemia, makes it particularly suitable as a therapeutic agent. Preliminary clinical findings have demonstrated that salvianolic acids (SA) has a positive impact on cerebral perfusion and neurological deficits in stroke patients, warranting further investigation. This review emphasizes SalA as a potential anti-inflammatory agent for the advancement of innovative therapeutic approaches in the treatment of ischemic stroke.

## 1 Introduction

Ischemic stroke is the primary cause of mortality and long-term disability of adult, posing a major global public health challenge (1). Ischemic stroke can result in devastating consequences, including paralysis, speech impairment, cognitive decline, and emotional disturbances, greatly impacting the quality of life for individuals and their families. The prognosis of stroke is influenced by various factors, including the severity of the initial ischemic insult, the extent of the resulting brain injury, and the body’s inflammatory response (2–4). Neuroinflammation plays a pivotal role in the pathobiology of ischemic stroke, exacerbating the damage to brain tissue and impeding the recovery process (5, 6). Despite advancements in acute stroke treatment due to mechanical thrombectomy and thrombolytic drugs, the available pharmacological interventions have shown limited efficacy in mitigating the long-term consequences of ischemic brain injury (7, 8). Neuroprotective agents have been a central area of investigation for nearly half a century (9), with the goal of safeguarding the brain from damage and fostering recovery. Despite numerous efforts, the success rate of these drugs in clinical trials has been limited. Of the more than 114 global clinical trials conducted, only a select few have managed to substantiate the efficacy of neuroprotective drugs (10), highlighting the profound scientific and clinical challenges in the field. The complexity of the underlying pathological processes, including the intricate interplay between ischemia and inflammation, has posed a significant challenge in the development of effective stroke therapies. In the face of these limitations, the exploration of natural products as potential therapeutic candidates has gained attention in the field of stroke research (11–13). Certain natural compounds, such as those derived from medicinal plants, have demonstrated promising neuroprotective and anti-inflammatory properties, offering potential avenues for improving stroke outcomes (14–17). These natural products may provide a complementary or alternative approach to conventional pharmacological interventions, potentially targeting multiple pathways involved in post-stroke neuroinflammation and facilitating recovery.

Salvia miltiorrhiza, also known as red sage or Danshen in Chinese, is recognized in traditional Chinese medicinal herb for its properties that enhance blood circulation, alleviate blood stasis, and improve microcirculation (18, 19). Among its bioactive constituents with a well-defined chemical structure, Salvianolic acid A (SalA), (2R)-3-(3,4-dihydroxyphenyl)-2-[(E)-3-[2-[(E)-2-(3,4-dihydroxyphenyl)ethenyl]-3,4-dihydroxyphenyl]prop-2-enoyl]oxypropanoic acid, has been identified as a particularly effective agent that possesses anti-inflammatory and antioxidant properties, as well as the ability to modulate the integrity and functionality of the blood-brain barrier (BBB) (20, 21). In line with these properties, SalA holds significant promise for the treatment of ischemic stroke, with its therapeutic mechanisms and functional targets potentially converging with the cascade of post-stroke neuroinflammation (22, 23). This review focuses on the pathophysiology of post-stroke neuroinflammation and summarizes the multifaceted pharmacological of SalA, particularly focusing on its immunomodulatory role in the treatment of ischemic stroke.

### 1.1 The inflammatory cascade in ischemic stroke

Ischemic stroke leads to interruption or reduction of cerebral blood flow, leading to a depletion of energy in the ischemic central region and subsequent irreversible neuronal necrosis (24). Disruptions in glucose and energy metabolism within the ischemic penumbra result in diminished Na^+^/K^+^-ATPase activity, subsequently perturbing ion homeostasis (25). Cell membrane depolarization causes Ca^2+^ influx and thus increases intracellular calcium concentrations, which induces an excessive release of the neurotransmitter glutamate (26, 27). The binding of glutamate to the receptors increases entry of Ca^2+^, thus causing mitochondrial dysfunction and inducing necrosis, which promotes cytotoxic oedema and inflammation in the surrounding tissue (28). Following a cerebral infarction, activated microglia release vasoactive mediators and pro-inflammatory cytokines such as interleukins (ILs) and tumor necrosis factor-α (TNF-α), which promote significant leukocyte infiltration and initiate neuroinflammation. These inflammatory cells also stimulate the generation of reactive oxygen species (ROS), leading to oxidative stress and further exacerbating the inflammatory reaction (29). Additionally, oxidative stress triggers the production and activation of matrix metalloproteinases (MMPs), leading to the degradation of tight junction proteins (TJPs) in endothelial cells and compromising the integrity of the BBB (30). This disruption allows toxic substances from the blood and peripheral immune cells to enter the affected brain regions, exacerbating brain edema and neuroinflammation.

Collectively, ischemic stroke triggers a cascade of pathological mechanisms that lead to neuronal damage, with inflammation playing a central role. SalA emerges as a potential modulator of the post-stroke neuroinflammation. This intervention has the potential to induce positive outcomes through the reduction of ROS and pro-inflammatory cytokines, ultimately leading to potential improvements in BBB integrity and mitigation of post-stroke neuroinflammation.

### 1.2 Pharmacokinetic characteristics of SalA in ischemic stroke

In order to preliminarily analyze the druggability of SalA, toxicological assessment and pharmacokinetic studies were conducted in non-diseased animals following oral or intravenous administration (31–33). The acute toxicity studies reported an LD50 of 1161.2 mg/kg in mice and identified a lethal dose range for Beagle dogs between 455–682 mg/kg through single intravenous SalA injections (31). In the subchronic toxicity study, intravenous SalA injections at 20, 80, and 300 mg/kg over four weeks in dogs revealed the no observed adverse effect level of 20 mg/kg, with higher doses associated with transient hepatic and renal effects and reversible thymus weight reduction (31). Notably, SAA showed no genotoxic effects in both the Ames test and the *in vivo* bone marrow micronucleus assay. These findings emphasize the importance of liver and kidney function monitoring during SAA administration, while its non-genotoxic nature supports its potential use in clinical settings and as a functional food ingredient.

Oral dosing in rats has illustrated a linear correlation between doses and the maximum plasma concentration (Cmax), which were measured as 31.53 µg/L for a 5 mg/kg dose, 57.39 µg/L for a 10 mg/kg dose, and 111.91 µg/L for a 20 mg/kg dose. However, despite its linear characteristics, the oral bioavailability of SalA was determined to be suboptimal, with a range of 0.39% to 0.52%. The limited permeability of SalA across the heterogeneous human epithelial colorectal adenocarcinoma cell (Caco-2) monolayer may account for this observed constraint in bioavailability, as indicated by an apparent permeability coefficient of less than 10^-6^ cm/s (32). In a distinct investigation utilizing beagle dogs, SalA exhibited prompt absorption subsequent to oral delivery, achieving maximum plasma levels within a two-hour timeframe. The absolute bioavailability was determined with a range of 1.47% to 1.84%, indicating quantifiable absorption via the oral route albeit at a diminished efficacy (33) Intravenous administration in rats delineated a mean residence time (MRT) of 2.91 hours and a half-life (t1/2) of 1.96 hours at a 5 mg/kg dose, indicating a relatively rapid systemic clearance (32).

Contrastingly, within the context of ischemic stroke, the pharmacokinetic profile of SalA is markedly altered due to the pathophysiological changes in the brain. A comparative pharmacokinetic analysis of SalA revealed that systemic circulation exposure to SalA was similar between sham controls and rats undergoing ischemia/reperfusion (I/R). However, there was a significant increase in brain exposure to SalA in the I/R group compared to the sham controls, with a fold change of 9.17, particularly evident at the early time point of 0.5 hours post-treatment (22). The increased permeability of the blood-brain barrier following I/R injury indicates that SalA may penetrate the compromised barrier and potentially provide neuroprotective benefits directly to brain tissue (22). Furthermore, metabolomic analysis demonstrated that SalA administration effectively mitigated metabolic disruptions associated with I/R injury, identifying 47 relevant metabolites in contrast to minimal metabolic changes observed in serum samples (22). Taken together, these findings suggest that SalA may serve as a promising therapeutic agent for treating ischemic stroke, given its pharmacokinetic characteristics that align well with the therapeutic requirements of the pathological condition of ischemic stroke.

## 2 Anti-inflammatory features of SalA in ischemic stroke

### 2.1 Inhibition of pro-inflammatory cytokines

**Figure 1:**
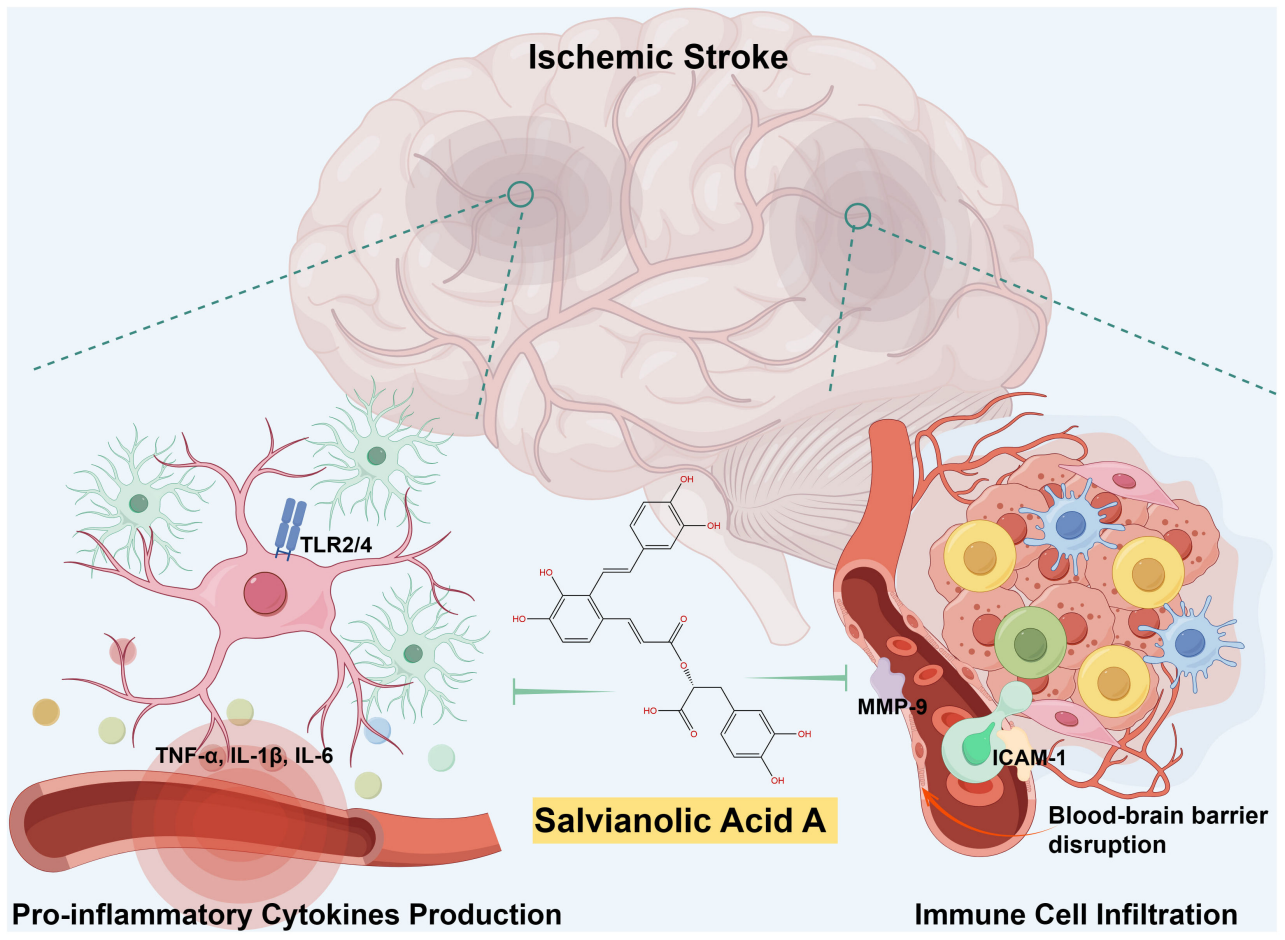
Schematic illustration of SalA’s anti-inflammatory effects during ischemic stroke. Following a cerebral infarction, activated microglia release vasoactive mediators and pro-inflammatory cytokines, thereby promoting significant leukocyte infiltration and initiate neuroinflammation. SalA, with its anti-inflammatory effects, specifically targets TLR2/4-mediated inflammatory pathways, the release of pro-inflammatory cytokines, MMP-9-mediated BBB leakage, and peripheral immune cell infiltrations. The synergistic effects of targeting multiple factors contributed to the improvement of post-stroke neuroinflammation recovery.

The depletion of cellular energy and subsequent necrosis of cells within the ischemic penumbra initiates a cascade of signaling events that stimulate inflammatory pathways, resulting in the secretion of inflammatory cytokines including IL-1β, IL-6, and TNF-α. These cytokines are pivotal in the development of ischemic stroke and contribute to the advancement of neuronal injury. Specifically, IL-1β and IL-6 play a crucial role in amplifying the inflammatory response, potentially leading to the activation of other immune cells such as microglia and astrocytes, which are intrinsic immune cells within the central nervous system (CNS). TNF-α has the ability to intensify inflammation by increasing the expression of adhesion molecules on endothelial cells, thereby promoting the migration of leukocytes into the ischemic area. SalA has shown significant efficacy in decreasing levels of IL-1β, IL-6, and TNF-α by inhibiting toll-like receptor (TLR) 2 and 4 signaling in microglia in both *in vivo* and *in vitro* models of ischemic stroke (34). Importantly, SalA downregulated TLR2/4 expression at both the mRNA and protein levels in the affected ipsilateral hemispheres, while no significant changes were observed in the non-ischemic contralateral hemispheres. Additionally, deficiency of TLR2/4 significantly decrease the anti-inflammatory efficacy of SalA, which revealed the TLR2/4-dependent inflammatory inhibition of SalA in microglia. The anti-inflammatory efficacy of SalA by inhibiting the TLR2/4 was also confirmed in hepatic ischemia-reperfusion (35) and heart failure (36). These findings suggest that TLR2/4 may represent prime targets for the anti-inflammatory therapeutics of SalA (left parts of **Figure 1**).

SalA has also been recognized to modulate key signaling pathways involved in the response to stroke-induced inflammation and apoptosis. SalA has been demonstrated to regulate the GSK3β/Nrf2/HO-1 pathway, which plays a crucial role in cellular defense mechanisms against oxidative stress. The protective function of heme oxygenase-1 (HO-1) is supported by its ability to inhibit the NF-κB signaling pathway, resulting in decreased TNF-α expression and subsequently mitigating inflammatory and apoptotic pathways (37). Furthermore, SalA has been demonstrated to have a direct action on the regulation of the NF-κB (38), a transcription factor crucial in immune response regulation and responsible for promoting the production of pro-inflammatory cytokines. Additionally, research has demonstrated that SalA exerts an influence on the PKA/CREB/c-Fos pathway (39), a signaling cascade that is integral in regulating various cellular processes, including cell viability and immune response. Through its multifaceted interactions, SalA potentially attenuates pro-inflammatory pathway and production of pro-inflammatory cytokines within the infarcted region, suggesting an anti‐inflammatory and neuroprotective properties against ischemic injury and therapeutic potential in the context of stroke.

### 2.2 Regulation of immune cell infiltration

The BBB serves as a crucial interface connecting the CNS with the peripheral circulation, consisting of endothelial cells, tight junction proteins (TJPs), and regulated by a complex interplay of astrocytes, pericytes, and other CNS cells (40). Under physiological conditions, the BBB enforces a stringent control over the paracellular and transcellular transport pathways, as well as a minimal level of transcytosis, thereby preventing the infiltration of potentially neurotoxic substances from the bloodstream into the CNS and maintaining its homeostasis (41). Serious BBB leakage induced by ischemic stroke causes peripheral immune cell infiltration, which exacerbates neuroinflammation and induces brain dysfunction and even death. Consequently, BBB protection is a standing topic in anti-inflammatory therapy of ischemic stroke.

Previous research indicated that SalA may protect against peripheral immune cell infiltration after ischemic stroke by inhibition of the CD11b/CD18 complex, intercellular adhesion molecule-1 (ICAM-1), soluble epoxide hydrolase (sEH), and granulocyte adherence (42–47). The modulation of these targets by SalA is instrumental in attenuating adhesion of peripheral immune cells to endothelial cells, a critical step in immune cells migration and subsequent inflammatory cascade. By inhibiting cell adhesion, SalA diminishes the inflammatory response associated with ischemic stroke. Moreover, SalA’s suppression of sEH enhances the protective effects of lipid mediators, which are vital for endothelial integrity and contribute to the mitigation of vascular injury and inflammation.

Matrix metalloproteinases (MMPs), such as MMP-2 and MMP-9, are implicated in the breakdown of TJPs like ZO-1, claudin-5, and occludin, which are critical for BBB integrity (48). The degradation of these TJPs by MMPs can lead to disruptions in BBB function (49). It was observed that I/R led to a significant decrease in the levels of TJPs, while treatment with SalA effectively prevented this decrease by suppressing the I/R-induced increase in MMP-9 (50). This protective mechanism of SalA is further supported by the significant reduction in Evans Blue extravasation, indicating a restoration of BBB permeability. The contributions of SalA on the BBB are further corroborated by its ability to reduce matrix metalloproteinase-9 (MMP-9) levels and increase tissue inhibitor of metalloproteinases-1 (TIMP-1) levels, both of which are essential for maintaining the structural and functional integrity of the BBB (50). Furthermore, the protective role of SalA toward the myocardial ischemia has also been attributed to its suppression of MMP-9 activity (51). Together, SalA decreases the migration of peripheral immune cells following ischemic stroke by reducing the expression of vascular adhesion molecules and inhibiting the breakdown of TJPs, resulting in vascular protection and ultimately reducing post-stroke neuroinflammation (right parts of **Figure 1**).

### 2.3 Limitations of current research on SalA

While the collective research efforts have shed light on the multifaceted anti-inflammatory actions of SalA in targeting various inflammatory pathways and mediators in ischemic stroke, there is a notable gap in our understanding of its precise molecular targets. Despite the promising findings that SalA modulates the activity of inflammatory signaling pathways and reduces the expression of inflammatory mediators, potentially through a TLR2/4 dependent mechanism (34), the precise molecular targets of SalA remain elusive. The anti-inflammatory effects attributed to SalA, although significant, are based on observations of downstream effects rather than direct interactions. It is postulated that SalA’s actions may be mediated through its binding to other proteins, thereby influencing downstream signaling pathways or secondary effects. However, the lack of direct evidence identifying the specific molecular targets of SalA limits our comprehensive understanding of its mechanism of action.

To elucidate the direct molecular targets of SalA, advanced techniques such as Activity-based Probes (ABPs) (52), which can provide insights into the enzymatic activity and binding preferences of the compound, are necessary. Furthermore, the application of Drug Affinity Responsive Target Stability (DARTS) analysis (53) could offer a means to assess the stability of the target protein in the presence of SalA, thereby indicating the compound’s binding affinity and specificity.

Additionally, the Stability of Proteins from Rates of Oxidation (SPROX) analysis (53) could reveal the impact of SalA on the oxidative stability of potential target proteins, which is particularly relevant given the oxidative stress associated with ischemic stroke. Cellular Thermal Shift Assay (CETSA) (54) and Thermal Proteome Profiling (TPP) (55) are other valuable methodologies that could be employed to assess the thermal stability of proteins upon SalA binding, offering further evidence of direct interactions.

The absence of such biophysical and biochemical analyses in the current research corpus means that while we can infer the anti-inflammatory and neuroprotective properties of SalA, we cannot conclusively demonstrate its direct molecular targets. This gap in knowledge is a significant limitation, as it impedes the full realization of SalA’s therapeutic potential and the development of more targeted and effective treatments for ischemic stroke.

## 3 Clinical efficacy and characteristics of salvianolic acid (SA) in acute stroke treatment

### 3.1 Clinical studies of SA in acute stroke treatment

Although there have been limited clinical studies specifically on SalA for ischemic stroke treatment, numerous studies have examined the therapeutic properties of salvianolic acids (SA) extract, a group of hydrophilic phenolic compounds sourced from Salvia miltiorrhiza, which contain defined SalA (56, 57). A recent clinical study was designed to evaluate the impact of salvianolic acid (SA) on improving blood flow to the brain in patients with ischemic stroke (57). The inclusion criteria mandated that patients be admitted within 72 hours of the onset of acute ischemic stroke symptoms, diagnosed with ischemic stroke confirmed by DWI, and have a Glasgow Coma Scale score above 5. Conversely, patients with a history of brain hemorrhage, very low consciousness levels, or allergies to contrast agents used in MRI were excluded to avoid complications. This study included a total of 159 patients, 85 in the SA group and 74 in the control group, with a mean age of approximately 60 years and an approximately equal gender distribution. The study was a randomized controlled trial, where patients were allocated to either the SA group or the control group. Notably, the study does not specify whether it was double-blinded, which is a methodological detail that could affect the results’ interpretation. The SA group received a daily dose of 130 mg SA for 14 days, administered intravenously dissolved in 250 ml of normal saline, in addition to standard therapy including aspirin and atorvastin. The control group received standard therapy and an equivalent volume of normal saline intravenously. The results indicated that patients treated with SA showed significant improvements in NIHSS and mRS scores at the 90-day follow-up, suggesting a potential neuroprotective effect of SA. Notably, the relative cerebral blood volume in patients with hypoperfusion improved markedly following SA treatment, as evidenced by perfusion-weighted magnetic resonance imaging (PWI) images. This suggests that SA may enhance perfusion in hypoperfused brain tissues, thereby improving neurological outcomes in acute stroke patients.

### 3.2 Expanding the clinical understanding of SalA: recommendations for future research and methodological considerations

In contrast to the well-defined pharmacological agents commonly used in Western medicine, the active constituents of natural medicines, such as SA and the individual components like SalA, are often not clearly identified. The underlying mechanisms by which these natural compounds induce neuroprotection remain largely elusive. the mixtures of multiple ingredients often overshadow lesser-known drug interactions, and pharmacokinetic interactions have been identified within the constituents of the Salvia miltiorrhiza extract (58, 59). A previous study demonstrated that Sal A increased the area under measured plasma concentration-time curve of denshensu ((R)-3-(3,4-Dihydroxyphenyl)-2-hydroxypropanoic acid) and salvianolic acid B and substantially decreased their clearances, possibly via the plasma protein binding replacement (59). In light of the identified pharmacokinetic interactions within the constituents of the Salvia miltiorrhiza extract, it is crucial for future studies to delve into the mechanisms of these interactions. Understanding the interplay between SA and other medications will be paramount in ensuring patient safety and optimizing the efficacy of SA treatment protocols. Moreover, in recognition of the complexity of SA which comprises a suite of compounds including SalA, future clinical investigations should specifically focus on the use of pure SalA in monotherapy settings. This targeted approach is designed to yield a more precise elucidation of SalA's intrinsic therapeutic effects, unencumbered by the potential interactions or influences of other SA components. This approach will provide a comprehensive understanding of SalA's therapeutic potential as an adjunct therapy, enhancing our knowledge of its role in the overall treatment strategy for ischemic stroke without compromising the standard of care.

Furthermore, it is noteworthy that the existing randomized controlled trials involving SA have predominantly enrolled Chinese patients (56). This demographic limitation raises questions regarding the generalizability of our findings to other ethnicities and populations. To address this, it is essential that forthcoming research endeavors to include diverse patient cohorts from various geographical regions. Such inclusivity will not only validate the universal applicability of SA (or SalA) but also uncover any potential variations in therapeutic responses across different populations.

In summary, while SA has demonstrated promise in the treatment of ischemic stroke, a more expansive and rigorous clinical research agenda is warranted. This includes broadening the scope to encompass larger and more diverse patient populations, employing multicenter study designs, implementing double-blind methodologies, and incorporating assessments of inflammatory biomarkers. Such an approach will not only bolster our comprehension of SA (or SalA)’s therapeutic potential but also pave the way for more efficacious treatment strategies for individuals afflicted with ischemic stroke and its sequelae.

## 4 Conclusion

In summary, Salvianolic acid A (SalA) has been recognized as a multifaceted therapeutic agent showing considerable promise for the treatment of ischemic stroke. The anti-inflammatory properties of SalA, which involve the suppression of pro-inflammatory cytokines, regulation of crucial signaling pathways, and direct effects on endothelial cells, suggest a potential neuroprotective role that could yield significant clinical advantages for individuals suffering from stroke. The pharmacokinetic profile of SalA, particularly its enhanced brain exposure following I/R injury, indicates a good fit for the therapeutic demands of stroke treatment. Clinical studies, albeit limited, have demonstrated promising findings on the effectiveness of SalA in enhancing neurological outcomes and cerebral perfusion in individuals with acute stroke. Further targeted clinical trials are required to fully understand the therapeutic capabilities of SalA and to establish the most effective treatment protocols. The incorporation of SalA into existing strategies for treating ischemic stroke may provide a supplementary method to traditional pharmacological interventions, potentially resulting in enhanced patient outcomes and quality of life.

## Author contributions

HY: Conceptualization, Data curation, Formal analysis, Investigation, Methodology, Resources, Software, Writing – original draft, Writing – review & editing. MI: Methodology, Writing – review & editing, Project administration, Data curation, Formal analysis, Investigation, Resources, Writing – original draft. SZ: Methodology, Resources, Software, Writing – review & editing. YS: Data curation, Investigation, Writing – original draft, Software, Visualization. JC: Writing – review & editing, Project administration, Supervision. HQ: Investigation, Project administration, Supervision, Writing – review & editing, Conceptualization, Formal analysis, Funding acquisition, Methodology. SY: Data curation, Visualization, Writing – original draft, Conceptualization, Formal analysis, Funding acquisition, Investigation, Methodology, Project administration, Supervision, Writing – review & editing, Resources, Validation.
